# Much has changed in the last decade except overall survival: A Swiss single center analysis of treatment and survival in patients with stage IV non-small cell lung cancer

**DOI:** 10.1371/journal.pone.0233768

**Published:** 2020-05-29

**Authors:** Till Wallrabenstein, Jessica Del Rio, Arnoud J. Templeton, Martin Buess

**Affiliations:** 1 Division of Internal Medicine, University Hospital Basel, Basel, Switzerland; 2 Faculty of Medicine, University of Basel, Basel, Switzerland; 3 Division of Medical Oncology, St. Claraspital, Basel, Switzerland; MD Anderson Cancer Center, UNITED STATES

## Abstract

**Background:**

Molecular therapies for cancers with targetable driver mutations and immunotherapies have revolutionized treatment of non-small cell lung cancer (NSCLC) during the last decade. International treatment guidelines began integrating targeted therapies in 2009 and immunotherapies in 2015. The aim of this study was to examine whether the benefits described for these new therapies in pivotal phase III trials have been translated to a real world patient population.

**Patients and methods:**

Data from all consecutive patients diagnosed with stage IV NSCLC diagnosed at a community hospital in Switzerland between 2007 and 2018 were analyzed. Three groups of patients were compared, namely patients diagnosed before 2009 (group 1), between 2009 and 2015 (introduction of targeted therapies, group 2) and after 2015 (introduction of immunotherapies, group 3). The primary outcome was overall survival (OS). Time to treatment failure was a secondary outcome. Both endpoints were estimated using the Kaplan Meyer method and compared by log-rank test.

**Results:**

408 patients were included. Patient characteristics were similar in the three groups. Median OS in groups 1, 2, and 3 was 9.8 (95% CI, 6.2 to 13.4), 9.9 (95% CI, 7.6 to 12.1) and 8.6 (95% CI, 6.6 to 10.5) months, respectively (p = 0.5). Across groups patients treated with targeted- and immunotherapies had a significantly better outcome than those treated with chemotherapy or best supportive care (p<0.001). Nevertheless, OS remained unchanged between groups despite adequate molecular testing and integration of targeted- and immunotherapies. Over time, the patient population got more morbid with respect to tumor burden (p = 0.02) and co-morbidities (p = 0.02).

**Conclusions:**

While selected subgroups of patients may benefit from new therapies, outcome in this unselected population of patients with stage IV NSCLC treated in daily practice has not improved over the last decade.

## 1. Introduction

Lung cancer remains a major cause of cancer-related mortality. In the US alone, around 142.000 deaths will be attributable to lung cancer in 2019.[1] It has the highest mortality rate of all cancers, accounting for 23.5% of all cancer-related deaths. Nearly half of all patients with lung cancer are diagnosed with stage IV disease.[2]

Treatment of metastatic non-small cell lung cancer (NSCLC) has undergone significant changes over the last decade. Chemotherapy has been the standard treatment for many years based on the positive results of numerous randomized trials and meta-analyses which have compared chemotherapy to best supportive care (BSC).[3],[4] Tyrosine kinase inhibitors (TKI) have revolutionized the treatment strategy for the small fraction of patients whose tumors harbor oncogenic driver mutations. Progression-free survival (PFS) could be significantly improved for patients e.g. with EGFR-mutated[5] or ALK-rearranged lung cancers.[6] Osimertinib, a second-generation TKI for EGFR-mutated cancers has recently been shown to significantly prolong overall survival (OS) in comparison to standard EGFR-TKIs.[7],[8] In ALK positive cancers alectinib showed further improvement of PFS compared to first generation substances.[9] However the follow-up time is still too short to conclude about the effect on OS. New specific therapies that will hopefully prolong survival are currently in development for patients with cancers harboring other oncogenic driver mutations such as BRAF, RET, NTRK etc. The introduction of immune checkpoint inhibitors has similarly changed former treatment paradigms. Several randomized trials have shown an OS benefit of checkpoint inhibitors compared to chemotherapy alone.[10],[11], [12], [13],[14] While OS at 1 year was about 33% for chemotherapy,[4] it increased to over 80% for targeted therapies[9] and to over 70% for immune checkpoint inhibitors.[13] National comprehensive cancer network (NCCN) treatment guidelines reflect this revolution, integrating TKIs since 2009 and immunotherapies since 2015, respectively. [15],[16] [17]

Here we analyzed the outcomes of patients with stage IV NSCLC treated in daily practice to explore the hypothesis that similar gains in survival would be observed with the integration of TKIs and immunotherapies.

## 2. Materials and methods

### 2.1. Patient selection, inclusion criteria and data acquisition

Our aim was to analyze outcomes of all patients diagnosed with stage IV NSCLC at one institution during a given time period, without any selection. To this end we consulted the general administrative database of our hospital, a community hospital with 230 beds in the northwest of Switzerland, to identify all patients with a C34 diagnosis according to ICD-10 (malignant neoplasm of bronchus and lung).[18] Subsequently, we screened all patients who were diagnosed between January 01, 2007 (earliest complete electronic patient records) and December 31, 2018. All consecutive patients with stage IV NSCLC were included. To avoid a stage shift over time, patients with proven pleural carcinomatosis or malignant pleural effusion that were classified as stage III according to the 6^th^ edition of the TNM classification for lung cancer were included, since they were treated as metastatic disease with palliative intent.[19] Patients with small cell lung cancer (SCLC), neuroendocrine tumors or pulmonary metastases of other tumor entities, as well as stage I-III NSCLC were excluded. Patients with incomplete data regarding histology and/or staging were also excluded. Minimal inclusion criteria were availability of a histological/cytological specimen and thoraco-abdominal computed tomography (CT) scan or positron emission tomography-CT (PET-CT).

The following data were collected from electronic patient records: date of diagnosis, age, sex, histology, PDL1 and molecular marker profile, localization of metastases, type and duration of treatment, localization and date of new metastases, time of death/time of last contact, relevant co-morbidity. Eastern cooperative oncology group (ECOG) performance status and smoking status was missing for a substantial number of patients.

Endpoints were recorded as of July 08, 2019, thus allowing for a minimal follow up of 180 days for all patients. The study was approved by the local ethics committee, Ethikkommission Nordwest-und Zentralschweiz (EKNZ, Switzerland) on November 28, 2018 (project ID 2018–02007). All patient data were fully anonymized before access for analysis. The ethics committee has waived the requirement for informed consent because of the retrospective nature of this study most patients had died before consent could be acquired.

In order to answer the question, whether treatment practice and survival has changed over time, we divided patients into three groups according to the date of diagnosis. As cut-off between time periods we chose the respective publication dates of new NCCN guidelines. Group 1 consists of patients diagnosed between January 01, 2007 (earliest electronic records) and December 20, 2009 (publication of NCCN guidelines by Azzoli et al.).[15] Group 2 consists of patients diagnosed between December 20, 2009 and October 20, 2015 (publication of NCCN guidelines by Masters et al.).[16] Group 3 consists of patients diagnosed between October 20, 2015 and December 31, 2018.

### 2.2. End points

The primary endpoint of interest was OS, defined as time from diagnosis until death with censoring at the date of last contact for patients lost to follow-up. Our secondary endpoint was time to treatment failure (TTF) with 1^st^ and 2^nd^ line of therapy. TTF was defined as time from treatment initiation to discontinuation of therapy for any of the following reasons: disease progression, toxicity, patient’s wish, physician’s decision, initiation of subsequent treatment line, or death/loss to follow up. Further secondary endpoints were comparison of treatment modalities of 1^st^ and 2^nd^ line treatment between groups, comparison of prognostic factors such as age, gender, histology, disease burden and co-morbidity between groups and a subgroup analysis of OS for patients receiving best supportive care. Disease burden was defined as total number of different organs affected by metastasis. Co-morbidities were defined as follows: Cardiovascular co-morbidities were defined as either heart failure of any cause and/or structural heart disease and/or previous stroke and/or peripheral artery disease and/or previous thrombosis and/or previous lung embolism. Pulmonary co-morbidities were defined as chronic obstructive pulmonary disease and/or emphysema and/or asthma and/or bronchiectasis and/or interstitial lung disease and/or asbestosis and/or previous lobectomy/pneumonectomy for any reason. Malignant co-morbidities were defined as any current or previous solid tumor entity other than NSCLC, however excluding malignant hematological conditions except for lymphomas.

### 2.3. Statistical analysis

Baseline characteristics were compared between the three patient groups by using Fisher’s exact test and Kruskal Wallis test for discrete and continuous variables, respectively. Distribution of treatment modalities (chemotherapy/molecular therapy/immunotherapy/best supportive care) between groups was also compared by Fisher’s exact test. Medians with 95% confidence intervals (95% CI) were estimated by using the Kaplan Meyer method and log-rank test was used to compare survival between groups. Median follow up time was calculated by using the reverse Kaplan Meyer method. Univariate Cox regression analyses were done to study the prognostic role of variables on the time-to-event endpoints. All statistical analyses were performed with SPSS version 25 (IBM Corp., Chicago, IL). No correction was applied for multiple statistical testing.

## 3. Results

### 3.1. Patient and disease characteristics

We screened electronic records of 1095 patients who were listed in the hospital database with a diagnosis of a malignant disease of the chest (C34). After exclusion of 209 patients with histologies other than NSCLC, 48 patients with missing data and 430 patients with stage I-III NSCLC, a total of 408 patients with stage IV NSCLC diagnosed between 2007 and 2018 were included in our study (S1 Fig). 94 of these patients (23%) were diagnosed before December 20, 2009 (group 1), 196 patients (48%) between December 20, 2009 and October 20, 2015 (group 2) and 118 patients (29%) between October 20, 2015 and December 31, 2018 (group 3).

Patient characteristics of the three groups are shown in Table 1. There were no significant differences between the three groups with regard to age, gender and histology. Across all groups, median age at diagnosis was 71 years. Median age of patients having received 1^st^ line therapy was 68 years and median age of patients having received 2^nd^ line therapy was 66 years. The majority of patients had adenocarcinoma (67%), followed by squamous cell carcinoma (16%) and other histologies (17%). Also the sites of metastasis were not significantly different between the 3 groups. Brain metastasis occurred with a frequency of 15%, 18% and 19%, respectively, in group 1, 2 and 3 (p = 0.75). However, regarding tumor burden, there were significantly more patients with metastases in 3 or more organs in groups 2 and 3 than in group 1 (p = 0.02). Also the number of patients with cardiovascular, pulmonary, and malignant co-morbidities increased significantly over time (p = 0.04, 0.001, and 0.02, respectively).

**Table 1:** Baseline and disease characteristics of all 408 patients with stage IV non-small cell lung cancer and of three subgroups.

Characteristic	TOTAL (n = 408)	Group 1 (n = 94)	Group 2 (n = 196)	Group 3 (n = 118)	p
Median age at the time of diagnosis *(range)*	71 *(37–94)*	69 *(44–87)*	71 (*46–94)*	72 *(37–89)*	0.40
Gender					
• Male *(%)*	218 *(53)*	47 *(50)*	108 *(55)*	63 *(53)*	
• Female *(%)*	190 *(47)*	47 *(50)*	88 *(45)*	55 *(47)*	0.72
Histology					
• SCC *(%)*	66 *(16)*	18 *(19)*	27 *(14)*	21 *(18)*	0.41
• Adenocarcinoma *(%)*	273 *(67)*	55 *(59)*	138 *(70)*	80 *(68)*	0.13
• Other, e.g. NSCC-NOS *(%)*	69 *(17)*	21 *(22)*	31 *(16)*	17 *(14)*	0.27
Molecular analysis					
• Molecular marker analysis available *(%)*	223 *(55)*	6 *(6)*	106 *(54)*	111 *(94)*	<0.001
• EGFR/ALK/ROS/BRAF-mutation *(%)*	53 *(13)*	2 *(2)*	33 *(17)*	18 *(15)*	<0.001
• PDL1>50%, no targetable mutation *(%)*	23 *(6)*	0 *(0)*	1 *(1)*	22 *(19)*	<0.001
Metastases					
• Pleura *(%)*	171 *(42)*	41 *(44)*	73 *(37)*	57 *(48)*	0.15
• Bone *(%)*	155 *(38)*	33 *(35)*	85 *(43)*	37 *(31)*	0.09
• Pulmonary *(%)*	114 *(28)*	21 *(22)*	55 *(28)*	38 *(32)*	0.28
• Hepatic *(%)*	80 *(20)*	16 *(17)*	42 *(21)*	22 *(19)*	0.66
• Central nervous system *(%)*	72 *(18)*	14 *(15)*	36 *(18)*	22 *(19)*	0.75
• Adrenal *(%)*	65 *(16)*	11 *(12)*	32 *(16)*	22 *(19)*	0.37
• Other *(%)*	75 *(18)*	16 *(17)*	34 *(17)*	25 *(21)*	0.67
Tumor burden					
• 1–2 sites of metastasis *(%)*	320 *(78)*	83 *(88)*	149 *(76)*	88 *(75)*	
• 3 or more sites of metastasis *(%)*	88 *(22)*	11 *(12)*	47 *(24)*	30 *(25)*	0.02
Relevant comorbidity					
• Cardiovascular *(%)*	218 *(53)*	40 *(43)*	108 *(55)*	70 *(59)*	0.04
• Pulmonary *(%)*	104 *(25)*	20 *(21)*	39 *(20)*	45 *(38)*	0.001
• Malignant *(%)*	79 *(19)*	9 *(10)*	43 *(22)*	27 *(23)*	0.02

Patients in group 1 were diagnosed 01/2007–12/2009, patients in group 2 were diagnosed 01/2010–10/2015, and patients in group 3 were diagnosed 11/2015–12/2018.

As expected, there were significant differences between the three groups regarding the availability of molecular biomarkers. Oncogenic driver mutation-status (EGFR, ALK, ROS and BRAF) was available only for a minority of patients in group 1 (6%). In comparison, 54% and 94% of patients in group 2 and group 3, respectively, had undergone molecular diagnostics (p<0.001). The number of patients with a known targetable mutation increased from 2% to 17% and 15% during the three time periods defining the groups (p<0.001). PDL1 status was tested on a regular basis since 2017. PDL1 status at the time of diagnosis was available for 79 of 118 patients (67%) in group 3. 19% of all patients in this group had a high PDL1 status (≥50%) without also harboring a targetable mutation.

### 3.2. Differences in survival over time and according to treatment approach

**Fig 1:**
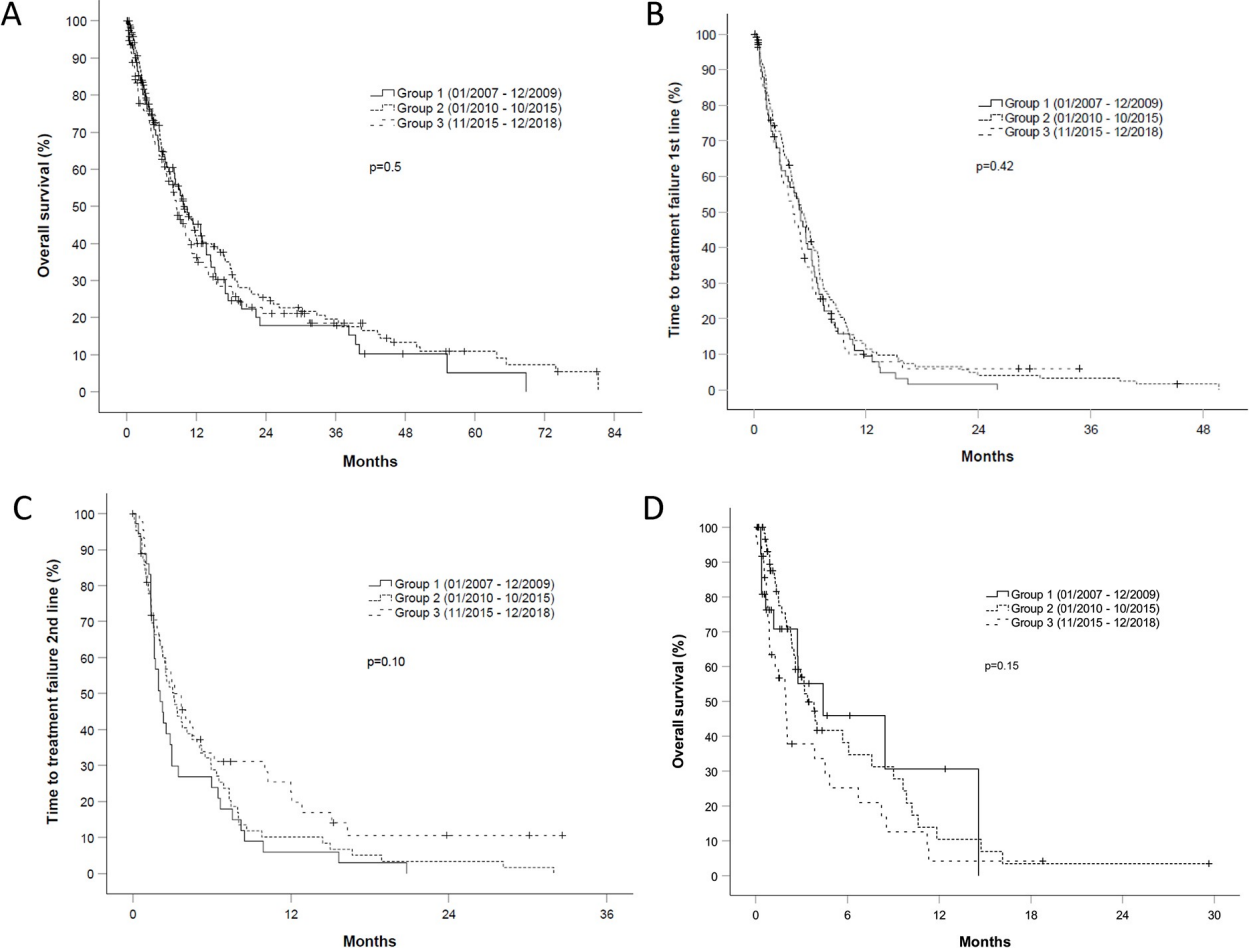
A-D. Overall survival (1A), time to treatment failure (TTF) of 1^st^ line therapy (1B), TTF of 2^nd^ line therapy (1C) and overall survival (OS) with best supportive care (1D) in patients with stage IV non-small cell lung cancer according to the date of diagnosis. Patients in group 1 were diagnosed 01/2007–12/2009, patients in group 2 were diagnosed 01/2010–10/2015, and patients in group 3 were diagnosed 11/2015–12/2018. All given p-values refer to comparison between all three groups. (A) Median OS in groups 1–3 was 9.8 (95% CI, 6.2 to 13.4), 9.9 (95% CI, 7.6 to 12.1) and 8.6 (95% CI, 6.6 to 10.5) months respectively (p = 0.5). (B) Median TTF of 1^st^ line therapy in groups 1–3 was 5.0 (95% CI, 3.7 to 6.4), 5.2 (95% CI, 3.9 to 6.4) and 4.2 (95% CI, 3.2 to 5.3) months respectively (p = 0.42). (C) Median TTF of 2^nd^ line therapy in groups 1–3 was 2.1 (95% CI, 1.3 to 2.8), 3.1 (95% CI, 2.1 to 4.1) and 3.2 (95% CI, 1.5 to 4.9) months respectively (p = 0.10). TTF of 2^nd^ line therapy was longer in the later groups as a trend, however this was not statistically significant. (D) OS of 126 patients receiving best supportive care: Median OS in group 3 was 2.0 months (95% CI, 1.3 to 2.6) as compared to 3.4 months in group 2 (95% CI, 2.3 to 4.5) and 4.4 months in group 1 (95% CI, 0 to 10.4). Patients in group 3 had a shorter OS as a trend (p = 0.15 for comparison between all three groups and p = 0.053 for direct comparison of patients in group 3 vs. patients in the earlier two groups).

A total of 270 patients had died after a median follow-up of 19.3, 30.3 and 25.1 months for groups 1, 2, and 3, respectively. Median OS in groups 1, 2, and 3 was 9.8, 9.9 and 8.6 months respectively (p = 0.5, Fig 1A). Confidence intervals and hazard ratios of all survival endpoints are summarized in Table 2. Median TTF of 1^st^ line therapy in groups 1–3 was 5.0, 5.2 and 4.2 months respectively (p = 0.42, Fig 1B). Median TTF of 2^nd^ line therapy in groups 1–3 was 2.1, 3.1 and 3.2 months respectively (p = 0.10, Fig 1C). TTF of 2^nd^ line therapy was significantly longer in group 3 in direct comparison to group 1 (HR 0.62, 95% CI 0.38–0.99, p = 0.045). We did not find any significant differences in specific reasons for treatment failure between the 3 groups (S1 Table). The subgroup of 126 patients receiving best supportive care had an OS of 4.4, 3.4 and 2.0 months respectively, showing a trend to a worse prognosis (p = 0.15 and p = 0.053 in direct comparison of group 3 with group 1 and 2, Fig 1D). A sensitivity analysis excluding patients who did not receive any systemic treatment showed a median OS of 10.4, 11.7 and 10.4 months for group 1, 2 and 3 respectively (p = 0.35).

**Table 2:** Overall survival (OS), time to treatment failure (TTF) of 1^st^ and 2^nd^ line therapy and survival in the subgroup of patients receiving best supportive care (BSC) in patients with stage IV non-small cell lung cancer according to the date of diagnosis.

Characteristic	TOTAL (n = 408)	Group 1 (n = 94)	Group 2 (n = 196)	Group 3 (n = 118)	p
OS (95% CI)	9.7 (8.2–11.2)	9.8 (6.2–13.4)	9.9 (7.6–12.1	8.6 (6.6–10.5)	0.50
• HR (95% CI)		1			
• HR (95% CI)			0.88 (0.65–1.19)		0.41
• HR (95% CI)				1.05 (0.75–1.48)	0.77
TTF 1st line (95% CI)	4.8 (4.1–5.5)	5 (3.7–6.4)	5.2 (3.9–6.4)	4.2 (3.2–5.3)	0.42
• HR (95% CI)		1			
• HR (95% CI)			0.83 (0.61–1.12)		0.22
• HR (95% CI)				0.99 (0.7–1.38)	0.94
TTF 2nd line (95% CI)	2.8 (2.3–3.4)	2.1 (1.3–2.8)	3.1 (2.1–4.1)	3.2 (1.5–4.9)	0.10
• HR (95% CI)		1			
• HR (95% CI)			0.82 (0.54–1.25)		0.35
• HR (95% CI)				0.62 (0.38–0.99)	0.045
OS of patients with BSC (95% CI)	3.2 (2.1–4.2)	4.4 (0–10.4)	3.4 (2.3–4.5)	2 (1.3–2.6)	0.15
• HR (95% CI)		1			
• HR (95% CI)			1.0 (0.52–1.92)		0.999
• HR (95% CI)				1.63 (0.82–3.22)	0.16

Median survival times are given in months. Hazard ratios (HR) are provided for group 2 and 3 in relation to group 1 as a reference of 1. Patients in group 1 (n = 94) were diagnosed 01/2007–12/2009, patients in group 2 (n = 196) were diagnosed 01/2010–10/2015, and patients in group 3 (n = 118) were diagnosed 11/2015–12/2018. BSC = best supportive care, CI = confidence interval, HR = hazard ratio, OS = overall survival, TTF = time to treatment failure.

**Fig 2:**
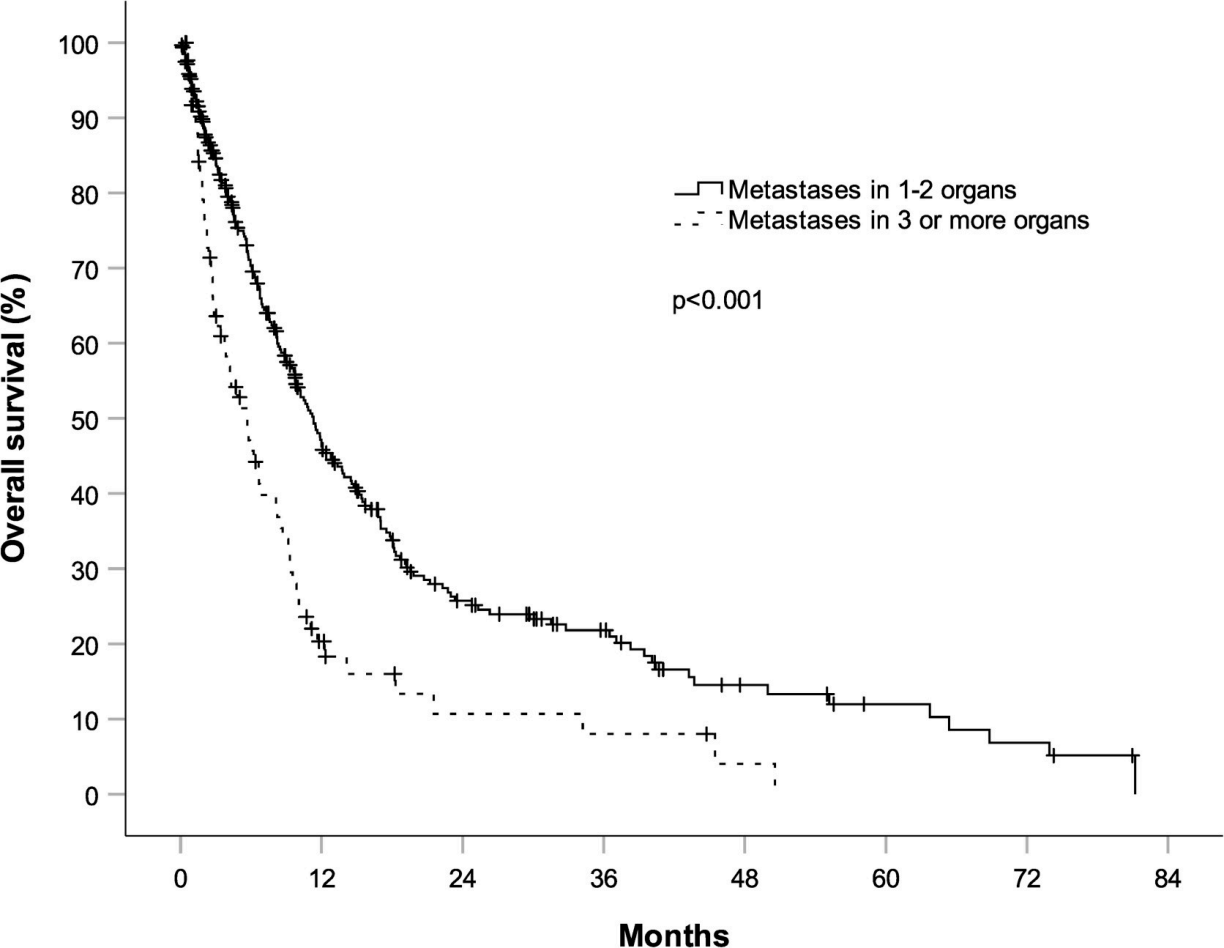
Overall survival (OS) according to number of organs affected by metastases in 408 patients with stage IV non-small cell lung cancer. The number of involved organs is significantly associated with survival. Patients with metastases in three or more organs had a median OS of 5.6 months (95% CI, 3.6 to 7.6) as compared to 11.3 months (95% CI, 9.6 to 13) for patients with metastases in only one or two organs (HR 1.95, 95% CI, 1.47 to 2.59, p<0.001).

Irrespective of therapy, we found that the number of organs affected by metastases is strongly associated with survival (Fig 2). In the overall study population, patients with metastases in 3 or more organs had a median OS of 5.6 months (95% CI, 3.6 to 7.6) as compared to 11.3 months (95% CI, 9.6 to 13) for patients with metastases in only 1–2 organs (HR 1.95, 95% CI, 1.47 to 2.59, p<0.001).

**Fig 3:**
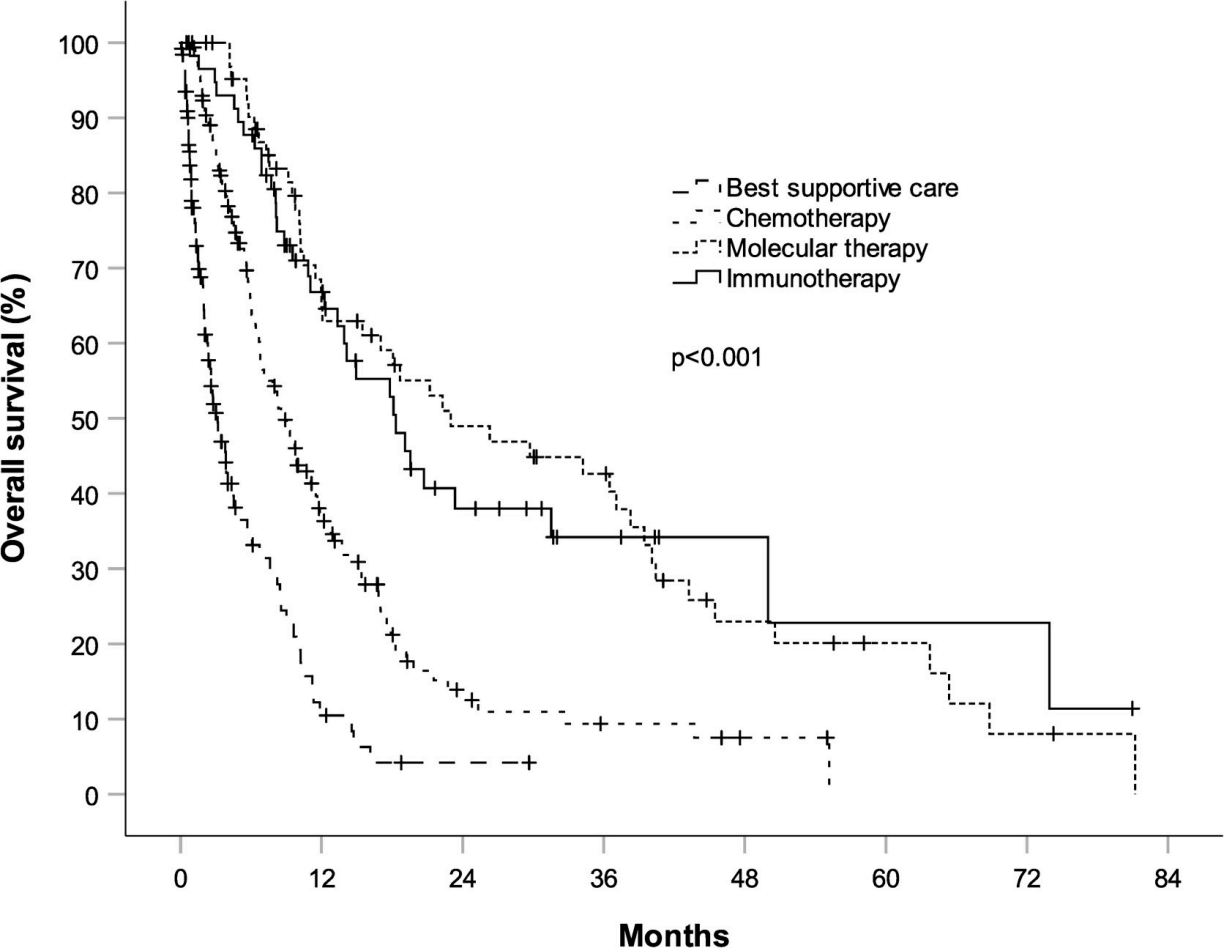
Overall survival (OS) from the time of diagnosis according to treatment modality in 408 patients with stage IV non-small cell lung cancer. 126 patients were unfit or unwilling for antineoplastic therapy and received best supportive care. 161 patients received chemotherapy. 64 patients have received molecular therapy and 57 patients have received immunotherapy at any time. Patients receiving chemotherapy had a significantly longer survival than patients receiving best supportive care (median OS of 8.7 months, 95% CI, 6.9 to 10.5 vs. 3.2 months, 95% CI, 2.1 to 4.2, p<0.001). Patients having received molecular- or immunotherapies had a significantly longer survival than patients having received chemotherapy (p<0.001). There was no significant difference in survival between patients having received molecular- or immunotherapies. Median survival for patients treated with immunotherapy was 18 months (95% CI, 12.8 to 23.8) and median survival for patients treated with molecular therapy was 23 months (95% CI, 8.4 to 37.6).

Median OS for patients treated with chemotherapy was 8.7 months (95% CI, 6.9 to 10.5) vs. 3.2 months (95% CI, 2.1 to 4.2) for patients receiving best supportive care, HR 0.40 (95% CI, 0.30–0.54), p<0.001 (Fig 3). Patients treated with either targeted- or immunotherapy at any time had a significantly better prognosis than patients treated with chemotherapy only, HR 0.41 (95% CI, 0.30–0.56, p<0.001). Median OS for patients treated with immunotherapy at any time was 18 months (95% CI, 12.8 to 23.8) and median OS for patients treated with TKIs at any time was 23 months (95% CI, 8.4 to 37.6). There was no significant difference in survival between patients treated with immunotherapy or TKI, HR 1.01 (95% CI, 0.69–1.73, p = 0.7).

### 3.3. Changes in treatment pattern over time

There was no significant difference between the three groups regarding the percentage of patients receiving systemic treatment. 29, 32 and 31 percent of patients in groups 1–3, respectively, were unfit or unwilling to receive any anti-tumor therapy and received best supportive care (p = 0.84). Thus, across groups, approximately 2/3 of patients received at least one line of systemic treatment. Approximately 1/3 of patients in each group (38, 33 and 37 percent in groups 1–3, respectively) received 2^nd^ line treatment (p = 0.54) and 14, 15, and 9 percent of patients in each respective group received 3^rd^ line treatment (p = 0.32).

**Fig 4:**
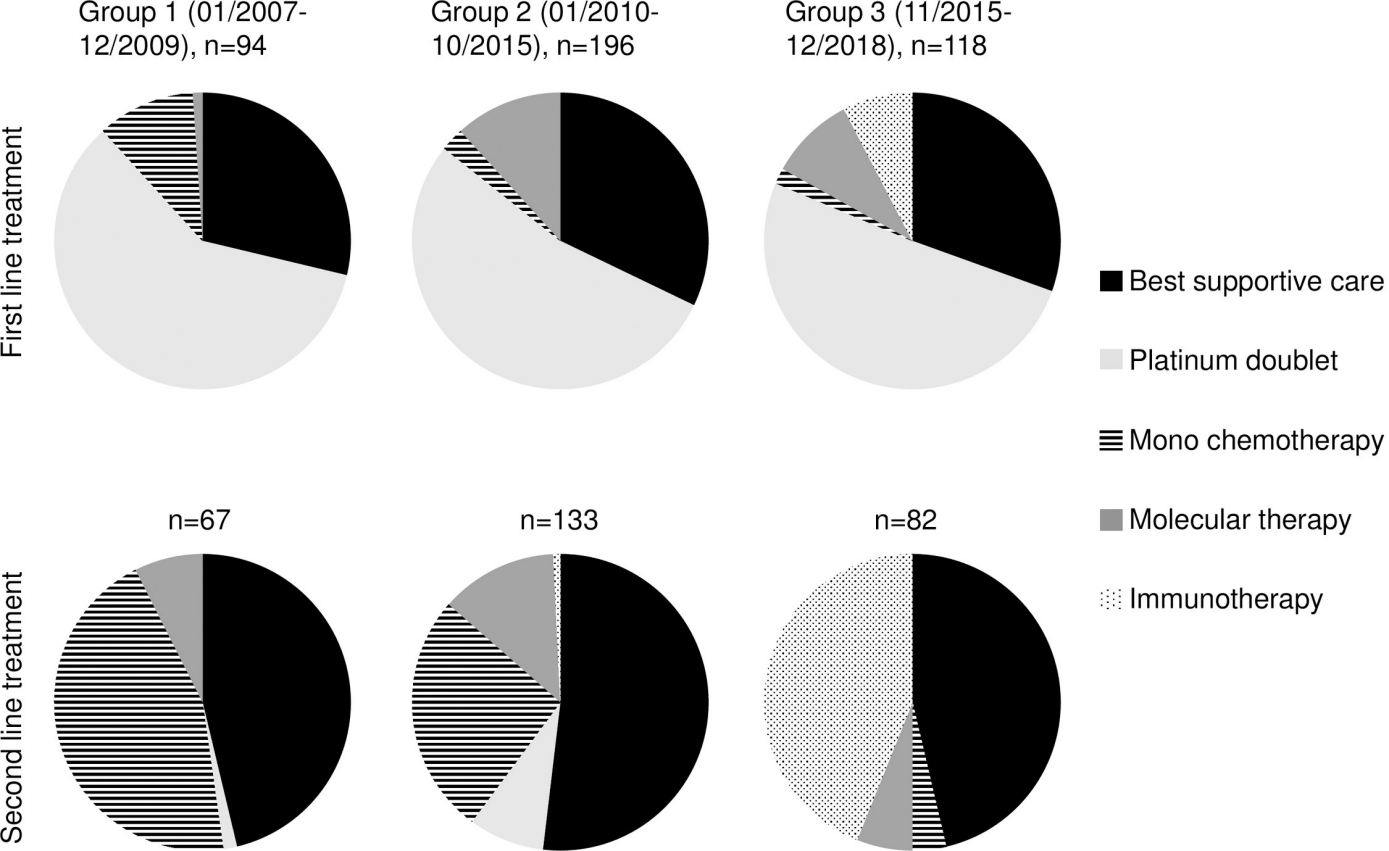
Distribution of treatment modality in 1^st^ and 2^nd^ line therapy of 408 patients with stage IV non-small cell lung cancer according to the date of diagnosis. Patients in group 1 (n = 94) were diagnosed 01/2007–12/2009, patients in group 2 (n = 196) were diagnosed 01/2010–10/2015, and patients in group 3 (n = 118) were diagnosed 11/2015–12/2018. In all three groups, about 2/3 of patients received antineoplastic 1^st^ line therapy and 1/3 received best supportive care. Only patients having received a 1^st^ line therapy were evaluated regarding a second line therapy.

We observed significant changes with regard to treatment modality over time, both in the 1^st^ and in the 2^nd^ line setting (Fig 4 and S2 Table). Across all three groups a majority of patients received platinum-based doublet chemotherapy as 1^st^ line therapy. The percentage of patients receiving mono-chemotherapy in the 1^st^ line decreased significantly from 15% in the first group, 4% in the second group to 2% in the third group (p = 0.004). As expected, the percentage of patients treated with molecular agents (1%, 17% and 13% respectively, p = 0.003) and immunotherapy (0%, 0% and 8% respectively, p<0.001) in the 1^st^ line increased significantly. We found that across all three groups 81% of patients with a known molecular target (EGFR, ALK, ROS or BRAF) received a TKI, 91% if considering only those patients who received any cancer specific treatment. 93% of treated patients with a known positive PDL1 status (≥50%) were treated with immunotherapy, the majority of them in the 1^st^ line.

We also found significant differences between groups regarding 2^nd^ line therapy. The majority of patients in group 1 and 2 received mono-chemotherapy as 2^nd^ line treatment. In contrast, 93% of patients in the third group received either a checkpoint inhibitor or–in the presence of a targetable mutation–a TKI. All in all, significantly more targeted- and immunotherapies were used in the later groups. The percentage of patients having received immunotherapy at any time has increased from 0% in group 1 to 43% in group 3 (p<0.001).

## 4. Discussion

Against our hypothesis of improved outcomes in recent years following the introduction of targeted therapies and immunotherapies, OS has essentially remained unchanged in patients with stage IV NSCLC treated during the last decade in our institution. This was despite broad access to and significant increase in use of immuno- and molecular therapies over time. What might explain this observation?

First we investigated whether patients were treated according to recommended standards. In all three groups about 1/3 of patients did not receive anti-neoplastic treatment. This number is within the usual range of other real world reports (41–79%).[20],[21],[22] We could show that most patients have received state of the art diagnostics. 94% of patients in group 3 have received molecular diagnostics and 67% PDL1 status testing. Similar studies have shown testing rates for PDL1 in the range of 24–50% and for EGFR/ALK in the range of 59–68%.[23],[21] Tumors were not only tested, but also treated according to molecular marker profile and PDL1 status with a strong permeation. In total, 91% of treated patients with a known molecular marker actually received a TKI and 93% of treated patients with a known PDL1 score >50% actually received immunotherapy, the majority in the 1^st^ line. 82% of patients in group 3 receiving 2^nd^ line therapy were receiving immunotherapy and 11% were receiving TKI.

Second, we compared survival of our patients to the survival of patients from other real world datasets and to results of randomized trials. Previous real world retrospective studies have shown significant improvements of OS for patients with stage IV NSCLC when comparing patients diagnosed in the late 1990s with the early 2000s.[24],[25],[26] To our knowledge, only few comparable studies have looked at real world changes of treatment patterns and survival up to recent years.[23],[27] These studies have found a small but statistically significant improvement of survival. However, these studies have included only patients who have received 1^st^ or 2^nd^ line treatment, excluding patients receiving best supportive care. Only one comparable study from Japan has included an unselected population of patients with stage IV NSCLC. However, this study included patients with significant epidemiological differences compared to our population. There was a much higher rate of cancers with targetable mutations, likely to benefit more from the introduction of TKI.[28] With a sensitivity analysis excluding patients who did not receive any systemic therapy we have shown that there was no significant and no clinically relevant improvement in OS over time in our population.

Although we could not establish a longitudinal improvement of survival, our real world data compares favorably to some large randomized trials. Schiller et al. have reported a median OS of 7.9 months (95% CI, 7.3 to 8.5) for patients receiving platinum based doublet chemotherapy as 1^st^ line treatment.[4] More recently Gandhi et al. have reported a median OS of 11.3 (95% CI, 8.7 to 15.1) months in their control arm with patients receiving platinum based chemotherapy in the 1^st^ line.[13] In comparison, OS from the beginning of 1^st^ line therapy was 11.1 months (95% CI, 8.9 to 13.3) in our overall population. Shepherd et al. have reported a median OS of 7.5 months for 2^nd^ line therapy with docetaxel in the TAX317 trial.[28] In comparison, OS from the beginning of 2^nd^ line therapy was 8.1 months (95% CI, 5 to 11.1) in our overall population.

Having found that OS in our unselected patient population compares favorably to randomized chemotherapy trials, we then looked closer at immunotherapies. Checkmate 017 and 057 have reported OS of 9.2 (95% CI, 7.3 to 13.3) and 12.2 (95% CI, 9.7 to 15.0) months for nivolumab as 2nd line therapy in squamous cell and non-squamous cell NSCLC respectively.[10],[11] Given that 82% of patients in our latest group have received immunotherapy in the 2^nd^ line (73% thereof have received nivolumab), our assumption was that survival would improve in this group. However, this was not the case. OS from the beginning of 2^nd^ line therapy was 7.2 months (95 CI, 2.5 to 12) in this group. These results still seem to be within the range of the numbers reported in Checkmate 017 and Checkmate 057, given that we are reporting for an unselected patient population. However, the question remains, why we did not find a longitudinal improvement between groups 1–3.

Third, we speculated whether demographics might have changed for patients in the later groups. Might these patients have a worse prognosis regardless of treatment modality? For each of the three time periods our study has included **all** patients with a definitive diagnosis of stage IV NSCLC, based on histology and complete staging with either thoraco-abdominal CT or PET-CT. We have shown that indeed patient demographics have changed towards a higher patient morbidity in the later groups. We have speculated that this might be because of hope invested in the new treatment options, which have recently become available and which are perceived to be more tolerable and possibly more effective than chemotherapy. If a patient was clearly unwilling or unfit to undergo chemotherapy in the past, there was no reason to enforce an affirmative diagnosis including histology and full staging. This perspective seems to have changed because such patients might still be considered fit and willing to undergo immuno- or targeted therapy. Thus we perceive a trend towards more definitive diagnostics, even for patients with a higher morbidity.

To support this interpretation we have re-evaluated the patient characteristics of groups 1–3. Our patient population seems to be representative regarding median age and distribution of histologic type as compared to other real world studies.[21] Median age at the time of diagnosis, gender, histology and the sites of metastasis remained unchanged over time (Table 1). Of note, also sites with an especially unfavorable prognosis, such as brain metastasis did not show a significant difference in distribution between the 3 groups. We compared the number of organs harboring metastases. Significantly more patients in the later groups had 3 or more different organs involved by metastasis (Table 1), which correlated with a worse survival (Fig 2). This association of survival with number of organs involved by metastasis in stage IV NSCLC has recently also been reported by other investigators and can be a valuable clinical prognosticator for daily practice.[29],[30],[31] It likely corresponds with reported differences of 5 year survival between stage IVA (10%) vs. stage IVB lung cancer (0%).[32] Furthermore, we demonstrated that significantly more patients in the later groups had cardiovascular, pulmonary and other malignant co-morbidities (Table 1).

This study also has limitations. First, this was a retrospective analysis with manual data extraction and entry, potentially leading to a risk of selection bias. We tried to account for this by selecting all consecutive patients treated in our institution during a defined period of time. Second, important prognostic information (e.g. ECOG performance status and smoking status) was not available for most patients which did not allow for adjustment of outcomes.

Our hypothesis was that more patients would receive systemic treatment in the later groups due to the newly available treatment options. However, we have observed that the proportion of patients receiving antineoplastic therapy has remained stable over time (about two thirds). We have interpreted this in support of our assumption that the overall patient population is being inflated by more morbid and frail patients. More treatment options are available, more patients seem to get diagnosed, and yet the same percentage of patients receives antineoplastic treatment. This indicates that most of those patients with a higher morbidity who might not have received definitive diagnostics in the past, are actually not receiving any anti-tumor therapy but best supportive care. Thus, the subgroup of patients who are receiving best supportive care seems to become especially inflated. In fact we have observed that OS got worse over time for patients receiving best supportive care as a trend (p = 0.15 in comparsion of all three groups and p = 0.053 in direct comparison of group 3 vs group 1 and 2, Fig 1D). After all, these data suggest that despite a rapid change of treatment practice during the last decade, we are still in need for better therapeutic options for this large–and seemingly growing–fraction of patients with stage IV NSCLC who are currently still receiving best supportive care.

As of 2018 combined chemo-immunotherapy has become a standard of care in first line treatment of patients without targetable mutations and PDL1 expression <50%.[13,14] Most treated patients are now receiving immunotherapies up front, instead of in the second line, as was largely the case in the patient population analyzed here, because upfront chemo-immunotherapy was not yet available. A future study should integrate these patients in a separate group and hopefully this will translate to a survival benefit in the overall population.

## 5. Conclusion

We have demonstrated that OS of an unselected population of patients with stage IV NSCLC at a Swiss community hospital has not improved over the last decade although TKI and immunotherapies have been properly integrated into daily clinical practice. The overall patient population in the later groups embraces more patients with co-morbidities and higher tumor burden. Despite possible advances for subgroups of patients e.g. patients with targetable driver mutations or high PDL1 status, outcome in the overall population did thus not improve, most likely neutralized by an increasingly morbid patient clientele. These data suggest that expectations should be managed carefully when extrapolating patient outcomes from highly selected clinical trials to an unselected real world population.

## Supporting information

S1 Fig Patient selection and division of 408 patients with stage IV non-small cell lung cancer (NSCLC) into three subgroups according to the date of diagnosis. Electronic records of all patients with lung/chest tumors hospitalized at a Swiss community hospital between 2007 and 2018 were screened. Of these 1095 patients, 687 patients were excluded; 430 patients with stage I-III NSCLC, 209 patients with histologies other than NSCLC and 48 patients with missing data regarding histology or staging. A total of 408 patients with stage IV NSCLC diagnosed between 2007 and 2018 were included in our study. 94 of these patients were diagnosed 01/2007–12/2009 (group 1), 196 patients were diagnosed 01/2010–10/2015 (group 2), and 118 patients were diagnosed 11/2015–12/2018 (group 3).
(PDF)

S1 Table Reasons for treatment failure (TTF) of 1st and 2nd line treatment in 408 patients with stage IV non-small cell lung cancer according to the date of diagnosis. Patients in group 1 (n = 94) were diagnosed 01/2007–12/2009, patients in group 2 (n = 196) were diagnosed 01/2010–10/2015, and patients in group 3 (n = 118) were diagnosed 11/2015–12/2018. TTF was defined as time from treatment initiation to discontinuation of therapy for any of the following reasons: disease progression, toxicity, patient’s wish, physician’s decision, initiation of subsequent treatment line, or death/loss to follow up. Here we provide the exact numbers, how often treatment was discontinued for any of these reasons in the total population and in each group. Given percentages refer to the number of patients treated in each line and group.
(PDF)

S2 Table Number of patients treated and distribution of treatment modality in 1st and 2nd line treatment of 408 patients with stage IV non-small cell lung cancer according to the date of diagnosis. Patients in group 1 (n = 94) were diagnosed 01/2007–12/2009, patients in group 2 (n = 196) were diagnosed 01/2010–10/2015, and patients in group 3 (n = 118) were diagnosed 11/2015–12/2018. Given percentages of each therapy line refer to the total number of patients per group. Given percentages of treatment modalities refer to treated patients per group. Significantly less mono chemotherapies and more immunotherapies were applied over time in both 1st and 2nd line treatment. ^a^ 6 of these 60 patients were concomitantly receiving immunotherapy (chemo-immunotherapy) in the 1st line.
(PDF)
