# Supplementary figures and images for: Much has changed in the last decade except overall survival: A Swiss single center analysis of treatment and survival in patients with stage IV non-small cell lung cancer
Source: PLoS One. 2020 May 29;15(5):e0233768. doi: 10.1371/journal.pone.0233768 (PMC7259780; doi:10.1371/journal.pone.0233768)

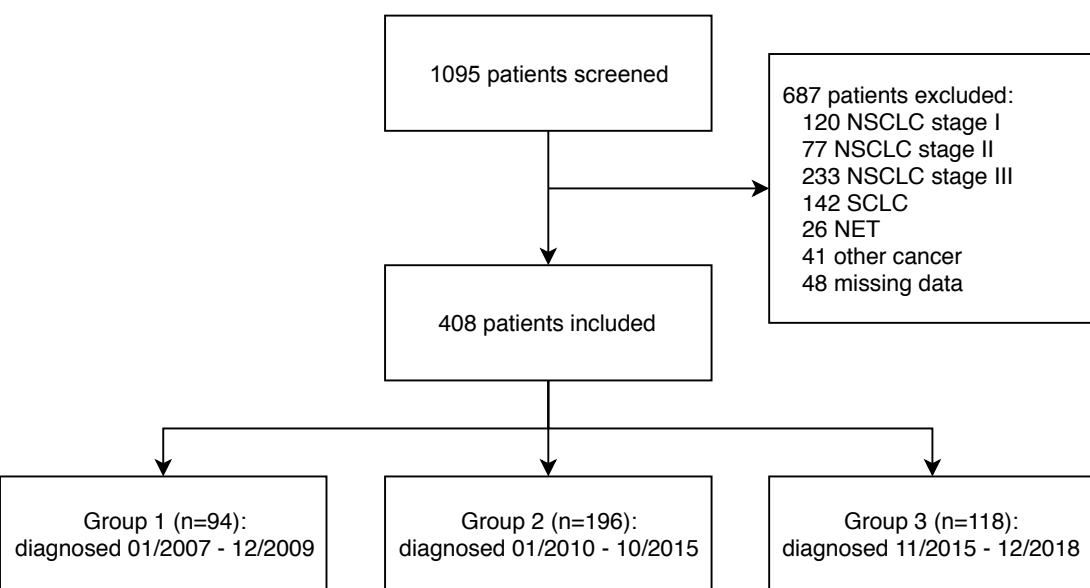

Supplement: S1 Fig — Electronic records of all patients with lung/chest tumors hospitalized at a Swiss community hospital between 2007 and 2018 were screened. Of these 1095 patients, 687 patients were excluded; 430 patients with stage I-III NSCLC, 209 patients with histologies other than NSCLC and 48 patients with missing data regarding histology or staging. A total of 408 patients with stage IV NSCLC diagnosed between 2007 and 2018 were included in our study. 94 of these patients were diagnosed 01/2007–12/2009 (group 1), 196 patients were diagnosed 01/2010–10/2015 (group 2), and 118 patients were diagnosed 11/2015–12/2018 (group 3). (PDF) [file pone.0233768.s001.pdf]
