# Supplementary material for: Much has changed in the last decade except overall survival: A Swiss single center analysis of treatment and survival in patients with stage IV non-small cell lung cancer
Source: PLoS One. 2020 May 29;15(5):e0233768. doi: 10.1371/journal.pone.0233768 (PMC7259780; doi:10.1371/journal.pone.0233768)
Supplement: S1 Table — Patients in group 1 (n = 94) were diagnosed 01/2007–12/2009, patients in group 2 (n = 196) were diagnosed 01/2010–10/2015, and patients in group 3 (n = 118) were diagnosed 11/2015–12/2018. TTF was defined as time from treatment initiation to discontinuation of therapy for any of the following reasons: disease progression, toxicity, patient’s wish, physician’s decision, initiation of subsequent treatment line, or death/loss to follow up. Here we provide the exact numbers, how often treatment was discontinued for any of these reasons in the total population and in each group. Given percentages refer to the number of patients treated in each line and group. (PDF) [file pone.0233768.s002.pdf]

Characteristic	TOTAL (n=408)	Group 1 (n=94)	Group 2 (n=196)	Group 3 (n=118)	p
Patients receiving 1st line treatment	282	67	133	82	
• Disease progression *(%)*	107 *(38)*	27 *(40)*	53 *(40)*	27 *(33)*	0.54
• Toxicity *(%)*	12 *(4)*	0 *(0)*	7 *(5)*	5 *(6)*	0.11
• Patient's wish *(%)*	13 *(5)*	3 *(4)*	7 *(5)*	3 *(4)*	0.93
• Physician's decision *(%)*	28 *(10)*	9 *(13)*	12 *(9)*	7 *(9)*	0.54
• Initiation of subsequent treatment line *(%)*	72 *(26)*	19 *(28)*	33 *(25)*	20 *(24)*	0.84
• Death *(%)*	28 *(10)*	6 *(9)*	12 *(9)*	10 *(12)*	0.71
• Loss to follow up *(%)*	22 *(8)*	3 *(4)*	9 *(7)*	10 *(12)*	0.2
Patients receiving 2nd line treatment	144	36	65	43	
• Disease progression *(%)*	47 *(33)*	9 *(25)*	26 *(40)*	12 (*28)*	0.23
• Toxicity *(%)*	14 *(10)*	1 *(3)*	10 *(15)*	3 *(7)*	0.1
• Patient's wish *(%)*	4 *(3)*	1 *(3)*	1 *(2)*	2 *(5)*	0.81
• Physician's decision *(%)*	17 *(12)*	7 *(19)*	6 *(9)*	4 *(9)*	0.29
• Initiation of subsequent treatment line *(%)*	25 *(17)*	9 *(25)*	12 *(18)*	4 *(9)*	0.17
• Death *(%)*	25 *(17)*	7 *(19)*	7 *(11)*	11 *(26)*	0.12
• Loss to follow up *(%)*	12 *(8)*	2 *(6)*	3 *(5)*	7 *(16)*	0.12
