# Supplementary material for: Much has changed in the last decade except overall survival: A Swiss single center analysis of treatment and survival in patients with stage IV non-small cell lung cancer
Source: PLoS One. 2020 May 29;15(5):e0233768. doi: 10.1371/journal.pone.0233768 (PMC7259780; doi:10.1371/journal.pone.0233768)
Supplement: S2 Table — Patients in group 1 (n = 94) were diagnosed 01/2007–12/2009, patients in group 2 (n = 196) were diagnosed 01/2010–10/2015, and patients in group 3 (n = 118) were diagnosed 11/2015–12/2018. Given percentages of each therapy line refer to the total number of patients per group. Given percentages of treatment modalities refer to treated patients per group. Significantly less mono chemotherapies and more immunotherapies were applied over time in both 1st and 2nd line treatment. a 6 of these 60 patients were concomitantly receiving immunotherapy (chemo-immunotherapy) in the 1st line. (PDF) [file pone.0233768.s003.pdf]

**Characteristic**	**TOTAL (n=408)**	**Group 1 (n=94)**	**Group 2 (n=196)**	**Group 3 (n=118)**	**p**
Best supportive care *(%)*	126 *(31)*	27 *(29)*	63 *(32)*	36 *(31)*	0.84
First line treatment *(%)*	282 *(69)*	67 *(71)*	133 *(68)*	82 *(69)*	
• Platinum doublet *(%)*	221 *(78)*	56 *(84)*	105 *(79)*	60[a] *(73)*	0.32
• Mono chemotherapy *(%)*	17 *(6)*	10 *(15)*	5 *(4)*	2 *(2)*	0.004
• Targeted therapy *(%)*	35 *(12)*	1 *(1)*	23 *(17)*	11 *(13)*	0.003
• Immunotherapy *(%)*	9 *(3)*	0 *(0)*	0 *(0)*	9 *(11)*	<0.001
Second line treatment *(%)*	144 *(37)*	36 *(38)*	64 *(33)*	44 *(37)*	0.54
• Platinum doublet *(%)*	12 *(8)*	1 *(3)*	11 *(17)*	0 *(0)*	0.002
• Mono chemotherapy *(%)*	68 *(47)*	30 *(83)*	35 *(55)*	3 *(7)*	<0.001
• Targeted therapy *(%)*	27 *(19)*	5 *(14)*	17 *(27)*	5 *(11)*	0.29
• Immunotherapy *(%)*	37 *(26)*	0 *(0)*	1 *(1)*	36 *(82)*	<0.001
Third line treatment *(%)*	54 *(13)*	13 *(14)*	30 *(15)*	11 *(9)*	0.32
